# Cloud Infrastructures for *In Silico* Drug Discovery: Economic and Practical Aspects

**DOI:** 10.1155/2013/138012

**Published:** 2013-09-10

**Authors:** Daniele D'Agostino, Andrea Clematis, Alfonso Quarati, Daniele Cesini, Federica Chiappori, Luciano Milanesi, Ivan Merelli

**Affiliations:** ^1^Institute of Applied Mathematics and Information Technologies, National Research Council of Italy, via de Marini 6, 16149 Genoa, Italy; ^2^Istituto Nazionale di Fisica Nucleare, Italian Grid Infrastructure, via Ranzani 13/2, 40127 Bologna, Italy; ^3^Institute of Biomedical Technologies, National Research Council of Italy, via F.lli Cervi 93, 20090 Segrate (Mi), Italy

## Abstract

Cloud computing opens new perspectives for small-medium biotechnology laboratories that need to perform bioinformatics analysis in a flexible and effective way. This seems particularly true for hybrid clouds that couple the scalability offered by general-purpose public clouds with the greater control and ad hoc customizations supplied by the private ones. A hybrid cloud broker, acting as an intermediary between users and public providers, can support customers in the selection of the most suitable offers, optionally adding the provisioning of dedicated services with higher levels of quality. This paper analyses some economic and practical aspects of exploiting cloud computing in a real research scenario for the *in silico* drug discovery in terms of requirements, costs, and computational load based on the number of expected users. In particular, our work is aimed at supporting both the researchers and the cloud broker delivering an IaaS cloud infrastructure for biotechnology laboratories exposing different levels of nonfunctional requirements.

## 1. Introduction

The current technological improvement of molecular biology techniques results in a huge expansion of biological data, whose satisfactory management and analysis are a challenging task. In particular, the adoption of an adequate computational infrastructure is becoming too expensive, in terms of costs and efforts of establishment and maintenance, for small-medium biotechnological laboratories. 

The traditional approaches of exploiting grid computing or buying computational power from a supercomputing center, in fact, are likely to provide an insufficient possibility to customize the computational environment. For example, it is common in computational biology to make use of relational database and web-oriented tools in order to perform analyses, which are difficult to exploit without being the administrator of the server. Another problem is the incredible number of bioinformatics packages that are available in different programming environments (such as R, Perl, Python, and Ruby), which typically require many dependencies and fine-tuned customization for the different users. Moreover, a large computing center often requires buying a predefined offer (i.e., a minimum fixed amount of computing time). 

These are the reasons why the present trend in e-Science is to deploy more flexible infrastructures, such as the service oriented ones. Cloud Computing (CC) technology, in particular the Infrastructure as a Service (IaaS) solution, provides full-computational infrastructures as a service through internet without requiring long setup [1]. These platforms foster the exploitation of available services paying only for the time they are used and make the computational environment greatly customizable, thus improving flexibility. In fact, the IaaS model allows a very high degree of customization, as users are free to install new Virtual Machines (VM) or modify preconfigured ones, such as Cloud BioLinux [2] or CloVR [3], enabling also fast and possibly computational intensive analyses. Concerning performance, a virtual infrastructure of 17,024 cores built using a set of amazon elastic cloud computing EC2 (http://aws.amazon.com/ec2/) VMs was able to achieve 240.09 TeraFLOPS for the High Performance Linpack benchmark, placing the cluster at number 42 in the November 2011 Top500 list (http://aws.amazon.com/hpc-applications/). 

However, the current commercial cloud infrastructures present several drawbacks. The first one is that not all the Cloud providers are equivalent (http://www.businessweek.com/articles/2012-05-07/the-economics-of-the-cloud), and it can be difficult to evaluate which is the one that best fits the users' needs. Moreover, a factual limitation of most cloud infrastructures concerns the poor offer of Quality of Service (QoS) supplied. Generally, the only QoS feature granted by providers is the availability of a service which is as the closest as possible to the 24 × 7 model [4]. But granting availability is not sufficient for many biotechnological applications, which require non functional features that are more specific as a high level of security or resilience. Besides this, most of the public clouds support the execution of any kinds of applications and may not represent a cost-effective solution for researchers requiring complex analyses. Another issue is that the current IaaS solutions are not completely suitable for large-scale HPC scientific simulations [5] even if several research efforts aim at a better support (http://arstechnica.com/business/2012/05/amazons-hpc-cloud-supercomputing-for-the-99/, http://www.hpcinthecloud.com/). A possible answer to cope with these issues, particularly for e-Science applications, comes from solutions that leverage existing grid infrastructures to deploy cloud-like services. This solution takes advantage of a more rational and flexible usage of these huge heterogeneous infrastructures to supply scientific users with customizable and scalable access to grid resources [6].

An alternative approach to cloud services provision is envisaged by important analysts such as Gartner, who predicted an exciting opportunity for the figure of the Cloud Broker (CB) [7]. According to Gartner, a broker is any service company that, acting as an intermediary between users and providers of cloud services, offers its expertise in the evaluation of the proposals and in the subsequent adoption or development of new products based on them [8]. CBs do not represent a new construct since brokers are commonplace in service oriented architecture. They can be considered as a specialization but with the opportunity to fill the gap due to the lack of suitable QoS levels. New players as small medium enterprises in fact can enter the market as CB by offering both customized and more application-oriented brokering services, along with specific higher quality services delivered on their private clouds.

In this paper, we provide insights to research groups concerning the effectiveness of cloud computing with respect to their needs in terms of trade-off between economic aspects and higher flexibility. This discussion can be very useful also for service providers that would like to approach this application domain in order to understand necessity and requirements of this field.

The analysis is based on the use of our brokering system for hybrid IaaS clouds (i.e., composed of resources belonging to public and private cloud infrastructures), improved to support the execution of virtual machines also on grid infrastructures. We considered a Drug Discovery (DD) pipeline as case study, where the various operations are characterized by different requirements and computational load. The requested services are allocated to the public or to the private cloud infrastructures based on the type of customers' QoS expectations and on the workload of the private cloud resources, having the twofold goals of satisfying the higher number of user requests and maximizing the CB's revenue. In our opinion, this kind of analysis is of interest both for small laboratories of molecular biology, to understand what the market can provide and at which cost, and for ICT service provider to understand the requirements of the computational biology domain.

The paper is structured as follows.  Section 2 describes an example of pipeline of operations for DD, followed by Section 3 that reports related work.  Section 4 presents the brokering system.  Section 5 details the examined metrics and the simulation setup, while Section 6 presents and discusses results. In Section 7, some conclusions are drawn.

## 2. An Example of Drug Discovery Pipeline

Modern DD is characterized by the need to examine huge compounds libraries in short periods of time. The use of *in silico* approaches for the selection of the best drug candidates to study in wet labs is critical because it can dramatically reduce the cost of the experimental phase. Many economical efforts of pharmaceutical industries and small-medium laboratories acting in the field of biotechnology are currently invested in the preliminary DD process performed using computational approaches. On the other hand, thanks to the large availability of resources, programs, processes, and protocols, *in silico* DD procedures are becoming more reliable day after day. Numerous drugs were recently obtained using DD pipelines, including Dorzolamide for the treatment of cystoid macular edema [9], Zanamivir for therapeutic or prophylactic treatment of influenza infection [10], Sildenafil for the treatment of male erectile dysfunction [11], and Amprenavir for the treatment of HIV infection [12].

**Figure 1:**
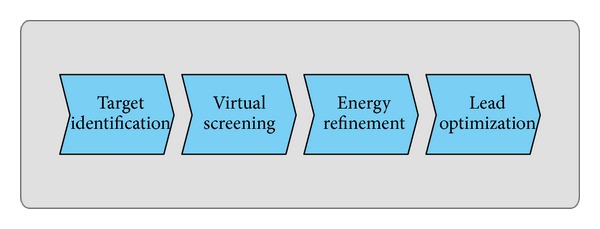
Principal operations of a typical drug discovery pipeline.

Each laboratory and each pharmaceutical industry have certainly refined their own specific DD pipeline, according to their possibilities and experiences. The procedures employed for DD are themselves well-kept secrets and can vary a lot in relation to the knowledge about the biological system, the computational and economic effort that can be supported. Although in this work we do not want to present a novel DD pipeline, a task that is widely discussed in the literature [13–17], we would like to exploit the experience matured in many national and international research projects in which experiments of DD have been performed, such as Bioinfogrid (http://www.bioinfogrid.eu), Italbionet (http://www.italbionet.it), Healthgrid, and Litbio (http://www.litbio.org/) [18–22], to discuss some economic and practical aspects of using modern cloud computing platforms for this purpose. Relying on our knowledge, we present an example DD pipeline that is composed of four principal steps, which in our experience constitute the backbone of every DD project and can be broadly summarized (see Figure 1) in (i) drug target identification using genomics and proteomics data, (ii) virtual high throughput screening of a large dataset of synthesizable compounds, (iii) energy refinement and analysis of the conformation, and (iv) lead optimization using quantitative structure-activity relationships.

### 2.1. Target Identification

By employing modern concept of biotechnology, the DD process nowadays starts with the identification of drug targets. Besides well-established targets, for which there is a good scientific understanding supported by a lengthy publication history, the current molecular biology technologies in the field of genomics, transcriptomics, and proteomics allow the identification of new targets. These approaches require greater research investment, but they are also the most promising ones. The combination of these “omics data” can be performed using a systems biology approach in order to enhance the possibility of identifying non trivial biomolecular mechanisms of action. 

Nonetheless, the creation of a systems biology model is a long task, which skilled researchers accomplish by hand, in particular when the model is quantitative and a mathematical description of the enzyme and metabolite dynamics is provided in function of their concentrations. For example, the analysis of the extended glycolytic pathway in normal and cancer cells has revealed that, besides the common employed drug targets, some enzyme of the close connected pentose-phosphate pathway can be targeted to fight the Warburg effect, a characteristic of cancer cells [23]. 

In detail, the optimization of the model against a specific cell condition consists in identifying some free parameters of the model in order to fit experimental data. This is a global optimization problem, usually addressed using genetic algorithms, which can be very time consuming, according to the number of parameters to identify. Many packages exist that provide suitable functions for this kind of optimization. A very common choice of free software is R-Bioconductor (http://www.bioconductor.org/), while among commercial solutions the use of MATLAB (http://www.mathworks.it/products/matlab/) with the support of the system biology toolbox should be cited.

### 2.2. Virtual Screening

The process of lead compound identification is the core of each DD experiment, and virtual screening is probably the most common structure based approach employed on this field. Virtual high throughput screening consists in performing a large scale docking experiment of a protein target against a large dataset of ligands with possible pharmacological activity. Many drugs have been developed starting from ligands identified using virtual screening, such as inhibitors of the serine protease [24], compounds for the seasonal flues [25], and compounds for neglected diseases [26]. Clearly, for the development of a virtual screening process, the structure of the target protein must be available to researchers. Short molecular dynamics can be used to relax the crystal structures or to optimize a comparative model of the protein target and sample different conformations of the target in order to improve the quality of results. 

A docking program must be chosen relying on many factors, such as the performance of the underlying algorithm and scoring functions with respect to the specific family of proteins. Common choices among the free available software are Dock [27] and Autodock [28], while among the commercial package we can find FlexX [29], Glide [30], and ICM [31]. The possibility to exploit commercial software on a preconfigured virtual machine can be an added value for a cloud provider specialized in the field of molecular biology. The dataset of ligand to screen must also be chosen with respect to the available knowledge of the protein of interest, although a common choice is the ZINC database [32], which collects 3.3 million chemical compounds annotated with biologically relevant properties (molecular weight, calculated Log *P* and number of rotatable bonds) from different vendors. These ligands have already been filtered according to the Lipinski rule of five [33], which evaluate the druglikeness of small molecule), and are classified in target specific subcategories for particular protein target in the DUD (http://dud.docking.org/) database which usually provides a starting point for the virtual screening. If a real ligand is known to bind a specific protein it can be interesting to test small molecules with quite similar characteristics; to accomplish this task another available molecule database is PubChem (http://pubchem.ncbi.nlm.nih.gov/) under the umbrella of National Institute of Health (NIH) which allows similar researches relying on the chemical structure of a molecule. Otherwise the ligand dataset can be designed by employing combinatorial chemistry methods.

The virtual screening step presents also some data management issues: although the size of the output is not huge, the number of compounds in analysis, the number of simulations performed, and the number of different conformations between the proteins and the ligands provided by the docking algorithm usually require the use of a relational database to be managed. 

### 2.3. Energy Refinement

Docking screenings are very useful for discarding compounds that clearly do not fit with the identified target. However, the effective selection of lead compounds is more difficult because docking software have well-known errors in computing binding energy in the range of some kcal [34]. Therefore, best compounds achieved through the virtual screening process usually undergo a protocol of energy refinement implemented using molecular dynamics [35]. This analysis consists in solving Newton's equations of motion for all the atoms of a protein, taking as boundary conditions the initial protein structure and a set of velocity having a Gaussian distribution. Indeed, by employing specific simulations schemas and energy decomposition algorithms in the postanalysis, molecular dynamics allow achieving more precise quantification of the binding energy [36]. Common techniques for energy estimation are MM/PBSA and MM/GBSA, which consist in the evaluation of the different terms that compose the binding energy. 

In particular, it is possible to account for the binding energy to the sum of molecular mechanical energies in the gas phase, solvation contribution, evaluated, for example, by using an implicit solvent model like generalized born or by solving the Poisson-Boltzmann equation, and the entropic contribution, estimated with normal mode analysis approximation.

### 2.4. Lead Optimization

Once a set of lead compounds has been identified, the last step of the DD process is the optimization of the compound in order to achieve molecules with improved potency compared to known ligands, if present (VS and ER steps), reduced off-target activities testing the obtained ligands against proteins related to the target, and physiochemical/metabolic properties suggestive of reasonable *in vivo* pharmacokinetics. The latter optimization is accomplished through the evaluation of chemical descriptors calculated employing quantitative structure-activity relationship (QSAR) analysis, in particular evaluating the ADME characteristics of the ligands through the Lipinski rule of five [33] and the subsequent ligand modification to improve the ADME properties.

Another application of QSAR analysis is the improvement of the final ligand set to be experimentally tested, relying on the physiochemical characteristics of previously selected ligands screened from different datasets, obtaining molecules with an estimated binding energy similar or improved compared to the previously selected [37, 38]. In this context, ligand modification, including the substitution of ligand functional groups to improve the solubility, for example, is one of the lead optimization strategies.

### 2.5. Computational Demand and Practical Requirements

The four steps of this exemplifying pipeline for DD present different characteristics in terms of Information and Communication Technology (ICT) requirements. The computational effort required for Target Identification (TI) analysis is nearly important, and both parallel and distributed approaches can be employed for the simulations. No specific requirement for the network is necessary because simulations are performed alone with no information exchange until the final statistical analysis. Nonetheless, the optimization of these mathematical models can take several days on a single machine, considering the number of parameters to estimate (typically from 10 to 50), the number of simulations required for each parameter (from hundreds to thousands), and the time needed for a single simulation, which can be in the order of 30 seconds. However, the characteristics of the parallel infrastructures are not very critical because simulations on the different parameters can be carried out in an independent way until the final collection of data, which makes this application also resilient to failures. Security issues are strictly connected with the data used for model optimization, which can impose some security constrains if information employed is sensible.

In the second step of the workflow, the Virtual Screening (VS), the size of the problem can result in an important computational effort. By using large-scale computational platforms, huge docking simulations can require a total computational time on a single core of many CPU-years [39]. Nonetheless, in real case studies, the number of ligands to screen is between 5,000 and 10,000 compounds. Screening thousands of ligands against a receptor, or even different conformations of it, usually takes some days depending on the parallelism degree available on the infrastructure. Security of this computation can be critical if ligands tested are from the pharmaceutical industries. For this reason, structural information of these ligands is often encrypted to avoid any possible hack. Although each single docking simulation is sensible to system failure, because typically no partial data are recorded until the computation is finished, the full task is composed of independent simulations, which are singularly performed. Virtual screening is an early phase analysis in the DD process so it is performed quite often even if the full pipeline only seldom comes to the very end. On the average, we can estimate that 75% of the TI steps are followed by a VS analysis, which requires a time proportional to the number of selected targets.

The computational effort required for the Energy Refinement (ER) analysis is probably the most relevant of the whole pipeline. In relation to the number of atoms involved in the simulation (also considering the presence of the explicit solvent, which is a common approach for molecular dynamics) and considering the number of steps at which the model is simulated (in our simulations from 5 to 300 nanoseconds), ER simulations can take weeks to compute also on parallel architectures. Moreover, about 10% of the starting dataset undergo the ER procedure; therefore, the number of complexes submitted to this step can be considerable. Molecular dynamics is well known to be very task intensive, and parallel computations are required to have results in a reasonable time. The parallelization paradigm on distributed clusters is usually based on the division of the simulated box assigning a set of atoms to each core. While employing a parallel cluster for parallelization, the use of high-performance network such as Infiniband is essential to improve performance; the use of Gigabit Ethernet usually prevents any benefit from using more than one server due to the high number of communications required to synchronize atoms at the box boundary. On the contrary, the use of a fast network results in a quite linear scalability at increasing the number of used CPUs. For example, by using 48 Xeon cores interconnected with a QDR Infiniband, it is possible to simulate a nanosecond of 250,000 atoms in 4 hours. The security required for this kind of applications is similar to the one of the previous step, and it is possible to set checkpoints in the computation in order to prevent the complete loss of data in case of failure. Generally, 50% of the DD analyses reach this operation, and it is common to run up to ten ER simulations at this stage. It is worthy to note that some molecular dynamics packages commonly employed for energy refinement, with particular concern to AMBER [40], can be also performed using GPGPU architectures. There is evidence (http://ambermd.org/gpus/) that exploiting the novel Kepler NVIDIA architecture a considerable scalability can be achieved, and also our tests are promising in this sense. Nonetheless, considering that many other programs used for molecular dynamics such as GROMACS [41], NAMD [42], and CHARMM [43] do not have the same scalability on these architectures, the exploitation of parallel computing is still widely used for this kind of simulations. From the cloud point of view, the use of GPGPU cards or the exploitation of an Infiniband connection presents similar problems, which means that a dedicated PCI device should be mounted to the virtual machine, with an additional cost, which should be valued according to the provided benefits. EC2 also provides some virtual machines equipped with NVIDIA Tesla “Fermi” M2050 GPUs. 

The Lead Optimization (LO) step is the most important because a positive result means the prosecution of the analysis in the wet lab, but it is executed a few times, due to project mortality along with the pipeline, and the less demanding one in terms of computational cost. Only 25% of the pipelines reach this operation that is performed on a number of compounds varying between 5 and 10, for an execution time that increases in a combinatorial way with the number of selected ligands. Programs used for this kind of analysis are often commercial (MacroModel (http://www.schrodinger.com/productpage/14/11/) and Medstere (http://syrris.com/batch-products/bioisostere-software)), although academic-free solutions are available (Discovery Studio (http://accelrys.com/products/discovery-studio/qsar.html)). As before, the possibility for a cloud service to provide access to this software, which can be also very expensive, can be an aspect to consider from the economic point of view. The time for this last step can be measured in hours, working on a workstation or a small server, which must be equipped with costly software and exploited by expert researches. 

From a computational and operative point of view, we summarized in Table 1 the functional (i.e., system configuration, type of required VM) and nonfunctional requirements along with the operation-specific features (i.e., input size, average arrival, and execution times) that characterize each step of the exemplified pipeline. Table 1 also lists some of the software solutions available as commercial or free packages for each operation in the pipeline. The example pipeline was implemented with a joint effort of the authors by installing these packages on the appropriate VM template (i.e., the one corresponding to the EC2 reference, as stated in Table 1) and measured the performance (i.e., service demand *μ*_*k*_) obtained by the virtualized execution of the software on real experiment data. The combination of system and operation values as well as of nonfunctional constraints is useful to provide a realistic, although approximate, account of the computational behavior of the exemplified pipeline model. Moreover, this set of data allowed us to analyze the performance and the costs of the proposed hybrid cloud broker in executing multiple pipelines, evaluated by means of the implemented simulator as discussed in the following sections.

We considered Amazon EC2 as the reference for the cloud providers because of the high quality of its infrastructure for parallel applications. On the basis of the operations' requirements, the Amazon EC2 candidate VM instances are presented in Table 2. As regards the prices, we considered the US East (N. Virginia) region in December 2012 today they can be different because they are highly volatile.

The Amazon's EC2 Compute Unit (ECU) (http://aws.amazon.com/ec2/faqs/#What_is_an_EC2_Compute_Unit_and_why_did_you_introduce_it) corresponds to the equivalent CPU capacity of a 1.0–1.2 GHz 2007 Opteron or an early-2006 1.7 GHz Xeon processor. In particular, the “C” instance type corresponds to an 8-core Xeon X5570n and D to a 16-core Xeon E5-2670. Considering also the ratio capabilities/price and the constraints of the previous tables, in our simulations we make use of the instance types C, D, and also E as composed of two instances of type D, as shown in Table 3 (see Section 5). It is possible in fact in Amazon to create clusters with high performance by defining “placement groups” (http://aws.amazon.com/hpc-applications/). Once the instance types were defined, we experimented the effective implementation of the operations considering the hardware configuration described in Section 5.2.3. The execution of the first three operations is carried out using a virtual cluster of VMs, because of their high level of data parallelism, as described before. The details of these configurations and the intervals of the possible execution times (i.e., the service demand times *μ*_*k*_) are shown in Table 1.

## 3. Related Work

Cloud computing in bioinformatics is presently used for many tasks from next generation sequencing analysis [44], data annotation [45], and genome processing [46, 47] to molecular modelling [48] and DD [1, 49]. In particular, DD is one of the user scenarios developed also within the virtual multidisciplinary EnviroNments USing Cloud Infrastructures (VENUS-C) EU-funded project (http://www.venus-c.eu/Pages/Home.aspx). VENUS-C aims at providing new cloud solutions for individual and small research groups across diverse disciplines through the development of a large cloud computing infrastructure for science and SMEs in Europe. In particular, this infrastructure provides a QSAR (http://www.isgtw.org/announcement/de-risking-drug-discovery-use-cloud-computing) service for creating models able to predict biological activity of compounds from their chemical structure. Such operation corresponds to the last step of the pipeline we presented in Section 2.

The growing interest for CC in bioinformatics [50] is due to the fact that it “can provide researchers with the ability to perform computations using a practically unlimited pool of virtual machines, without facing the burden of owning or maintaining any hardware infrastructure,” as said by the J. Craig Venter Institute (http://www.jcvi.org/cms/research/projects/jcvi-cloud-BioLinux/overview/) describing the motivation for their Cloud BioLinux image [2]. This can be considered the prosecution of the effort to develop suitable grid infrastructure for managing, integrating, and analysing molecular biology data. Starting from the experience in UK of myGrid (http://www.mygrid.org.uk/), which proposed a platform for distributed analysis and integration of bioinformatics data, other initiatives followed in France, such as IdeeB (http://idee-b.ibcp.fr/) GRISBI (http://www.grisbio.fr), and Renabi (http://www.renabi.fr/), in Germany with D-Grid (http://www.d-grid.de/) and MedGrid (http://www.medigrid.de/), and in Italy with LITBIO, LIBI, ITALBIONET, and Interomics (http://www.interomics.eu). At the moment, large EU funded projects are ongoing for the creation of infrastructures aimed at the analysis of omics-data through distributed and cloud infrastructures, such as Elixir (http://www.elixir-europe.org/), StratusLab (http://stratuslab.eu/), Contrail (http://contrail-project.eu/), and Eurocloud (http://www.eurocloudserver.com/about), which followed very well-known initiatives such as EMBRACE (http://www.embracegrid.info/).

There are also a number of private initiatives that aim at supporting research, in particular in bioinformatics and computational biology, using cloud Infrastructures. Examples are Era7 Bioinformatics (http://era7bioinformatics.com), DNAnexus (https://dnanexus.com), Seven Bridge Genomics (https://www.sbgenomics.com), EagleGenomics (http://www.eaglegenomics.com), MaverixBio (http://maverixbio.com/), and CRS4 (http://www.crs4.it/). Noteworthy, also large providers of molecular biology instrumentations, such as Illumina, and huge service providers, such as BGI, have CC services to support their customers.

A common situation for small-medium labs is in fact the periodical need to process large amount of data. As said in the introduction, the classical approach is to exploit the power of the clusters (or supercomputers) owned by their institutions (i.e., the university or research institute) or accessible through grid environments, exploiting partnerships and grants, or by paying the price for outsourcing to third party ICT providers. Each of these choices implies to modify the code to get it running on these resources, difficulties to have their software installed, and long waiting time in a scheduling queue. This is the reason why solutions relying on IaaS, where VMs are available to install whatever software (and version) needed by the research group, are highly desirable. Nonetheless, the mere availability of the most commonly used bioinformatics tools in a preconfigured VM is an added value, because they are quite a large number and hard to build and maintain.

An interesting solution is represented by the Worker Nodes on Demand Service (WNoDeS) [6], developed by INFN, which is able to dynamically allocate VM on a resource pool, in particular in the EGI grid computing environment. The key added value of this tool is represented by its smooth integration with the Local Resource Management System (LRMS); therefore, it is possible to exploit the same resource pool via the grid, local, and WNoDeS interfaces without the need to partition it. With respect to our previous works, we improved our brokering system in order to be able to interact with it. Although this tool is installed on a few INFN grid sites [51], which limits the size of the simulation that is possible to perform, it has a great potential. As regards the aspects related to the QoS requirements, they depend on the resource provider; therefore, for high-level request it is necessary to exploit commercial services different from the grid initiatives. 

The interest about providing ICT services in the field of molecular biology is great, as we can see, and this is only the top of the iceberg if we consider that CC is supposed to become of primary interest in the healthcare sector once the concerns related to security and privacy will be adequately solved. For these reasons, a sound evaluation of the economic aspects involved in working in this field is of paramount importance for a successful adoption of CC solutions for bioinformatics applications, and the analysis proposed in this paper goes in this direction. 

Within the VENUS-C project, an interesting analysis about the sustainability strategies was carried out [52]. According to that report, an e-Science cloud infrastructure, in order to be sustainable, has to (i) expand the participation of users within a research community and also to involve other research communities, (ii) simplify the access to the offered services and provide a customized support, and (iii) improve virtualization in order to improve consequently the availability, accessibility, efficiency, and cost-effectiveness of the platform.

However, while it is easy to quantify pros and cons in the adoption of a cloud solution for a single and possibly fast operation [1, 53], it is more difficult to develop a suitable forecasting method able to predict economic performances of cloud technologies, in particular for long running workflows as we did. In some cases, depending on the volume of data and computation to perform, operating on a cloud provider's infrastructure seems to be more expensive than providing the necessary ICT infrastructure in-house. As recognized in a technical report on CC published by the UC Berkeley Reliable Adaptive Distributed Systems Laboratory [54], “when all the costs of running a data centre are factored in, including hardware depreciation, electricity, cooling, network connectivity, service contracts, and administrator salaries, the cost of renting a data centre from a Cloud provider is marginally more expensive than buying one. However, when the flexibility of the cloud to support a virtual data centre that shrinks and grows as needed is factored in, the economics start to look downright good.”

On the market, there are many companies offering general-purpose cloud computing services (the public cloud) with many similar features, but it is hard to compare the value of these services. This is due to several motivations, but probably the truth is that vendors do not want a too straightforward comparison (http://www.informationweek.com/cloud-computing/infrastructure/why-cloud-pricing-comparisons-are-so-har/240001491). This is one of the reasons why a brokerage service for the discovery of available resources and the definition of service level agreements for their use is highly desirable, as recognized by Gartner, and in this case is of particular importance to smooth the issues related to e-Science clouds [55].

Several commercial and open source cloud broker platforms and tools support organizations in consuming and maintaining cloud services, being IaaS, PaaS, SaaS, or NaaS (network as a service), particularly when they span multiple providers (see Table 3). These offers generally “automates and accelerates the creation, launch, and management of all types of scalable cloud environments—whether public clouds, private cloud, virtual private clouds or hybrid clouds,” and may be provided on-premises, as SaaS or both solutions. More or less, all these tools grant QoS features like security, reliability, and resiliency allowing SLA mechanisms to enforce them. Some CB tools are more consumer or service provider oriented, while others may be employed by cloud providers or by cloud brokerage enterprises to start their own core businesses. The last three systems listed in Table 3 are component-based frameworks originated from EU FP7 projects.

As regards the brokering strategies, several proposals were presented in the literature, aiming at the design of efficient algorithms for the negotiation of the resources, as [56], at the minimization of energy consumption, or, on the other hand, at maximizing the efficiency of resources, as [57, 58]. Here, we focus on the evaluation of economic aspects; therefore, we evaluate three simple but meaningful strategies for the evaluation of the possible costs a laboratory has to pay and the revenue a CB can obtain.

To ground our proposal with real market offers, we evaluated the HPC features supplied by public cloud providers with respect to the requirements of the considered pipeline operations. Cloud computing infrastructures in general provide a good support to loosely coupled programs where processors work independently for long periods and exchange a limited amount of data [59]. However, Amazon EC2 has set up a higher standard for performance with its IaaS offer, providing a wider set of VM classes from micro (for low throughput applications with periodic bursts) to graphics processing units (GPUs) based, and presently it appears as the most advanced offer (http://arstechnica.com/business/2012/05/amazons-hpc-cloud-supercomputing-for-the-99/) also compared to Microsoft Azure [60]. For this reason, our analysis leverages on Amazon EC2's instance types and pricing models.

## 4. Hybrid Clouds Brokering System

**Figure 2:**
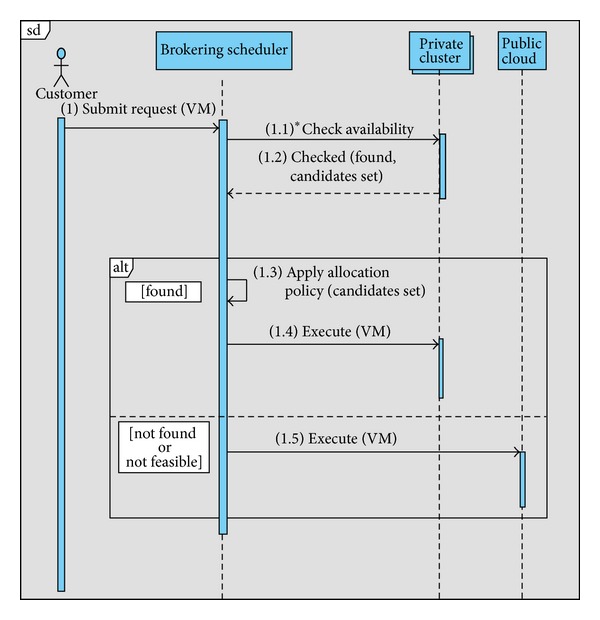
UML sequential diagram of a request for service execution in a hybrid cloud.

Both public cloud providers and the CB itself can supply the resources managed by a Hybrid Cloud (HC). The UML sequential diagram of Figure 2 highlights a typical and quite general scenario for HC. A customer requires the CB for one of the services it provides (e.g., TI, VS, ER, and LO), which are delivered by the CB through VMs equipped with the necessary software. The brokering scheduler allocates the VM to the private or public cloud zone depending on the availability of feasible private resources and according to service's computational demand and nonfunctional constraints. The CB offers different VM configurations for the services expressed in the form of some Service Level Agreement (SLA) templates depending on their requirements and price schemas (see Table 3). This differentiated offer is aimed at satisfying various kinds of nonfunctional requirements (e.g. security and privacy constraints, high bandwidth network, and resiliency to faults) as well as at presenting customers with an articulated portfolio of prices from which they can choose a tailored solution which best fits their needs. 

With the purpose of maximizing the number of satisfied users (by reducing their waiting time) along with the CB's revenue (by maximizing the execution of the VMs on its private cloud), the proposed brokering algorithm takes into account the characteristics of the requested services in terms of their temporal and hardware requirements, thus achieving a profitable use of its resources. In a mixed scenario with possible competing customers (i.e., having different QoS requirements), a too aggressive pursue of incomes may negatively influence the satisfaction of a significant part of them. Indeed, a naïve way to increase the total revenue is to maximize the in-house execution of all services. Such a greedy approach is generally hindered by two factors: the limited number of in-house resources and the specific requirements of each service that may force some requests to run preferably on the public zone and reserve private space to particularly critical ones (e.g. security constraints). It is therefore important to carefully analyze the way requests for service executions are better satisfied by in-house or by public resources. 

As discussed in Section 2.5, the services involved in the DD scenario are characterized by different functional and nonfunctional requirements. In particular, from Table 1, we see that each class of service may be requested to be executed on a secure and resilient system or be independent from this constraint. In the following, we call Private cloud Zone (namely, for short PZ) a request of the first kind and Any cloud Zone (namely, AZ) a request which can be indifferently executed on the private or the public zone. While AZ requests, for definition, can almost immediately be accepted, thus always meeting users demand, PZ ones have to be mandatorily allocated on the private resource and therefore may be subject of delays or, in the worst cases, rejections if not enough space is found for their allocation in a predefined amount of time.

Any allocation strategy, which strictly tries to satisfy the majority of PZ requests, could lead to a conservative, hence, less-profitable situation to reserve all in-house resources, thus renouncing to possible revenue by rejecting AZ request, even when there are free private resources. On the other side, an allocation mechanism that indiscriminately assigns AZ requests to private nodes increases the risk of missing or delaying PZ requests. By taking into account system utilization and the characteristic of each request, a viable allocation solution will properly use part of the private resources to run AZ services (aimed at increasing the total revenue), keeping however a reasonable amount of them free, ready to satisfy possible future PZ requests.

### 4.1. System Architecture

**Figure 3:**
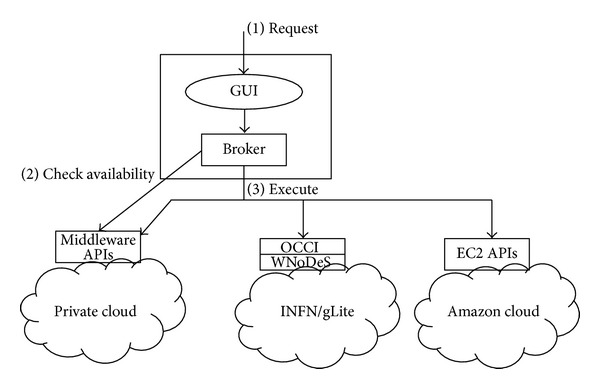
The brokering system architecture.

In Figure 3, the architecture of the brokering system we developed is shown. The user interacts with the tool via a web interface that allows specifying the service he/she needs (1). These parameters are sent to the Broker that interacts with the private cloud to obtain the resource status (2). This interaction is not possible, in general, with the public clouds, which only provide the ability to launch predefined VM templates and monitor them. It is also to be considered that such information would also be not necessary because usually public clouds have enough resources to satisfy most of the requests. The broker scheduler then decides where to allocate the request on the basis of the policies described in Section 5 that consider the status information, the type of service, and the required QoS and issues the launch command on the selected cloud using the proper API (3).

With respect to our previous works [61, 62], we improved our system in order to be able to interact with WNoDeS through the Open Cloud Computing Interface (OCCI) standard (The OGF Open cloud Computing Interface, (http://www.occi-wg.org/doku.php)), therefore we are now able in theory to exploit the power of Grid computing infrastructures for the execution of VMs. We say “in theory” because so far the tool is limited to a few EGI sites; therefore, we have to deal with public CC providers also for AZ requests. The hope is that initiatives similar to the aforementioned grid-based ones will support this attempt of convergence between grid and cloud computing. A further example is also provided by the Sci-Bus project (http://www.sci-bus.eu), which allows creating e-Science gateways for both the infrastructures.

### 4.2. Brokering Strategies

The multiple clusters of the HC belong to two *administrative zones*: the *private one*, directly operated by the CB, and the *public* one managed by commercial providers, amounting for *N* and *P* (with *N* ≪ *P*) physical servers, respectively. In the following, we characterize each server *s* by the tuple:

(1) capability(s) =〈tot_num_core,tot_amount_RAM,tot_amount_HD〉.

The brokering algorithms schedule job by applying heuristics based on different quota of private resources to execute the various service requests. The parameter *Q*, *Q* ≤ *N*, determines the private portion of servers dedicated to the execution of type PZ requests. 

Each request *req* of service execution is characterized by an execution time according to the class of service requested and possibly by a deadline. In particular, in the simulation we assumed that AZ requests are executed on the public cloud when a fixed deadline expires. Furthermore, according to its hardware requirements, *req* is mapped to a specific virtual machine described by the tuple:

(2) $$\begin{array}{c} \text{vm}\left(\text{req}\right)=\langle {\text{num}}_{\text{cores}},{\text{amount}}_{\text{RAM}},{\text{amount}}_{\text{HD}}\rangle . \end{array}$$

While we assumed that it is always possible to allocate requests to the public zone at any moment, only a subset of private ones is available, depending on its current workload. At any instant, the number of available resources for a node *s* executing *R* requests is given by

(3) $$\begin{array}{c} \text{available}\left(s\right)=\text{capability}\left(s\right)-\sum_{j}\text{vm}\left({\text{req}}_{j}\right)\;j=1,\ldots ,R, \end{array}$$

where ∑_*j*_vm(req_*j*_) is the addition on vectors and − is the subtraction operation on vectors.

The brokering scheduler allocates a VM to the private zone for an arriving PZ request *req* if (4) holds; elsewhere *req* is put in a waiting queue until an available server is found:

(4) $$\begin{array}{c} \exists \;s\in \text{private}\;|\;\text{vm}\left(\text{req}\right)\leq \text{available}\left(s\right). \end{array}$$

Moreover, for an AZ request to be allocated to the private zone, the following has to conjunctly hold with (4):

(5) ∑sj ∈ private  available(sj)>QN  ∗  ∑sj ∈ private  capability(sj).

Condition (5) checks if the whole amount of available private resources is actually greater than the quota of resources reserved only to PZ, determined by the parameter *Q* ∈ [0 ⋯ *N*]. Depending on the value of *Q*, the brokering algorithm allows depicting three allocation patterns, namely, zone strategies: feasible (*Q* = *N*), static reservation (0 < *Q* < *N*), and max occupation (*Q* = 0). With Feasible (FE), all private resources are dedicated to perform PZ requests only: that is, the *Q*/*N* ratio is equal to 1, and condition (5) is neither satisfied. According to Max Occupation (MO), no resource is used exclusively to perform PZ: *Q*/*N* = 0, and (5) is always true. Static Reservation (SR) strategy, instead, reserves a fixed quota of resources *Q* to execute PZ requests and lets *N*-*Q* resources free to execute the other kind of requests (i.e., AZ). As we discussed in Section 6, the choice of *Q* affects both CB's revenue and user satisfaction and strictly relates to the real system workload.

The adoption of each one of the three zone strategies (i.e., the value of *Q*) discriminates the behavior of the algorithm in allocating requests to the private or public cloud. Once a set of available resources has been found on the private zone, a second level of scheduling decision may apply to select the private servers eligible to allocate the scheduled service (see Figure 2).

## 5. Materials and Methods

To analyze the achievements of the brokering algorithm with respect to the three scheduling strategies FE, SR, and MO, we developed a discrete event simulator [63], taking into account the following description of the parameters and metrics involved in the simulated scenario.

### 5.1. Performance Metrics

The revenue of a CB is function of the service prices that, in the case of hybrid cloud, may include the cost of acquired resources from a public provider. Given a request to execute, for a period *t*, a service of type *k* (e.g., TI, VS, ER, and LO), a customer has to pay the CB the price *p*_*k*_:

(6) $$\begin{array}{c} p_{k}=B_{k}+t*C_{k}, \end{array}$$

where *B*_*k*_, the brokering service price, is the fee owed to the CB to handle the request, and it is due irrespectively whether the request is executed in-house or on the public cloud. The second term of *p*_*k*_ is the de facto provisioning price, proportional to the execution time *t*, and depends on the capabilities (e.g. number of cores, disk space, network connection, and so forth) required by each specific service, expressed by the hourly cost *C*_*k*_, associated with the virtual machine delivered. If the service is executed on the private cloud, the CB revenue is given by the sum of the two prices. Otherwise, the CB revenue is limited to *B*_*k*_ and the provisioning price is turned to the public cloud provider. The revenue of the CB to execute the request *j* on the server *i*, where the class of *j* is *k*_*j*_ and the execution time is *t*_*j*_, is

(7) revenueij=Bkj+tj∗Ckj, i∈private,revenueij=Bkj i∈public.

The (annual) total CB's revenue, for its brokering activity, accounts for all the *X*_*i*_ requests executed on each server *i* of the whole hybrid cloud (*i* = 1,…, *N* + *P*; *j* = 1,…, *X*_*i*_):

(8) $$\begin{array}{c} \text{revenue}=\sum_{i}^{}\sum_{j}^{}{\text{revenue}}_{ij}, \end{array}$$

where *X*_*i*_ is the throughput [64], that is, number of requests completed by each server *i*  (*i* = 1,…, *N* + *P*) of the hybrid cloud.

A second metric used to analyze performance behavior of the brokering algorithm is the system utilization *U*. We are obviously interested in the utilization of the private cloud, under control of the CB, which is obtained considering the busy time *U*_*i*_ of each private server (*i* = 1,…, *N*):

(9) $$\begin{array}{c} U=\sum_{i}U_{i}. \end{array}$$

Notwithstanding, for the kind of service considered, each request can be “unfold” into different VMs that can be allocated as soon as enough space is found; we measured the user satisfaction has the average waiting time *W*_*k*_ before a PZ request of execution is completely allocated to the private zone. Indeed, the AZ ones can be immediately satisfied on the public zone and also when executed on the private nodes, they wait for a maximum deadline in the worst case. As opposite PZ services may wait days, until a sufficient amount of private resources returns idle the execution of a complete pipeline, made of four PZ requests (as in the proposed example), requires an average waiting time *W*, *r* ∈ {TI, VS, ER, LO}:

(10) $$\begin{array}{c} W=\sum_{r}W_{r}. \end{array}$$

### 5.2. Simulation Setup

In this section, we describe the parameters used to perform the experiments, which are the arrival times and the average service times for the classes of requests, the prices we considered, and the composition of the private cloud infrastructure.

#### 5.2.1. Arrival Times and Service Demand Times

The values of the parameters used in the simulation are based on the information collected in many years of research activity within the bioinformatics group of the Institute for Biomedical Technologies. Moreover, relying on discussions carried out in the frame of Italian initiatives (http://www.iit.it/en/nasce-iddn-italian-drug-discovery-network.html, http://www.interomics.eu/) and considering market analysis performed by possible investors (www.iban.it/guidapratica.pdf), we make the hypothesis that a research group may perform up to 4-5 DD projects each year, and we considered the possibility of supporting up to 40 teams at European level (i.e., a total of 160 pipelines). As regards the arrival and the service demand times, we used synthetic workloads generated by statistical functions. The frequency of arrivals of the requests (*λ*_*k*_) of each class of service *k* ∈ {TI, VS, ER, and  LO} during the year is modeled with a uniform distribution, not privileging particular time ranges (e.g. daytime/nighttime) and month (e.g. weekdays/weekends). With respect to more sophisticated solutions [65], this choice is justified by the fact that some steps require execution times of several days, and it seems not particularly relevant to distinguish when they arrive. Also, the service demand times of the classes of requests (*μ*_*k*_) are uniformly distributed in the specific time-range of their class (e.g. TS requests lay between 16 and 160 hours), as defined in Table 1.

#### 5.2.2. Definition of Prices

This is the most important aspect of the simulationsetting step. Among the public cloud providers, Amazon EC2 provides suitable solutions for high performance computing applications, so we analyzed its pricing models (http://aws.amazon.com/ec2/pricing/) for their adoption in our simulation. Three are the models proposed: on-demand instances, reserved instances, and spot instances. We disregard the last one, designed on a bid-based approach on spare Amazon EC2 instances for time-flexible and interruption-tolerant tasks, focusing on the first two of them. Let us consider the most powerful VM instance type among those provided (http://aws.amazon.com/ec2/instance-types/), the Cluster Compute Eight Extra Large (see Table 2). If we consider the on-demand instance model, designed for users not paying a yearly subscription, each instance costs $2.4 per hour, which is $1,728 for a month and more than $20,000 for a year. If the VM will be used for the whole year, the lowest price is achievable using the heavy utilization reserved instances formula, and it is of $3,163 plus a subscription of $5,000. However, it has to consider that customers have to pay a number of subscriptions equal to the number of VM they want to run at once, and that they will pay the full price even if they do not use the VM for some periods. In this last case, customers could consider the medium utilization reserved instances formula; the cost per hour becomes $0.54 and the annual subscription $4,146, but they will pay just for the effective VM execution. We want to point out that the simulated prices based on Amazon EC2 ones are close to the prices exposed by the other public cloud providers for the same kind of VMs provided. 

In our opinion, all the Amazon pricing models are too expensive for an e-Science environment; therefore, in our simulation, we considered a cheaper scenario keeping however the same subdivision into two classes of users: *sporadic* and *frequent*. According to the definitions of Section 4 we assumed that sporadic customers would submit AZ requests, while frequent users will submit the PZ ones. Therefore, the first class of users will pay the Amazon price for on-demand instances plus the brokering service *B*_*k*_ that the simulator computed as a 5% of the provisioning price. This solution is reasonable as the brokering algorithm is always able to allocate their requests (at least) on the public cloud on the basis of the scheduling strategy and the private cloud load. This class is intended for research groups performing a limited number of requests and that do not require high-level QoS. For frequent users, or users requiring a high level of security and resiliency, an annual subscription, not related to the number of VMs they actually execute, is foreseen and the less expensive price is applied. The amount of the subscription fee will be not considered in this work because its definition should take into account many factors related not only on transferring the operational costs of a cloud infrastructure to customers, but also on a balancing between the forecasted usage of the cloud of a research group with respect to the others. The importance of setting the fee properly is clear also considering that the experimental results showed an average cost around $1,100 to execute a complete pipeline for frequent user while, for the same pipeline, sporadic users will pay around $ 10,000. From a research group point of view, the clear evaluation of the prices offer is strictly related to the number and the type of requests it is going to submit and is certainly a key factor in deciding to choose a cloud solution instead of more traditional ones (as discussed in Section 3). The other important aspect to consider besides the mere price examination is the higher possibility to customize the execution environment and to avoid the initial setup and periodic maintenance of an in-house ICT infrastructure. For sporadic customers, the brokering service *B*_*k*_ owed to the CB is counterbalanced by the fact that they can take advantages of its intermediation with respect to a public cloud provider, because the former grants to manage all the technical issues to put in execution and monitor the required VMs. Moreover, a CB is able to provide well-tuned VM according to SLA subscriptions that can also be equipped with nonopen source software. Table 3 reports hardware configurations for each type of VM instance (see Section 2.5) and the differentiated hourly prices offer, supplied to PZ and AZ customers for the execution of each pipeline operation. 

#### 5.2.3. Private Cloud System Configuration

Considering the infrastructure currently available in the context of our research institutes and taking into account the possibility of dedicating part of this infrastructure to an external service, the configuration of the private cloud infrastructure, we adopted in our simulation, is a cluster composed of 10 diskless nodes equipped with four 8-core Xeon processors, 128 GB of Ram, linked together via an Infiniband network, that can be also used for high-speed communication inside the virtual machine using PCI passthrough (http://www.linux-kvm.org/page/How_to_assign_devices_with_VT-d_in_KVM) approach. The same method can be used to share the NVIDIA K20 cards, providing the possibility of exploiting these architectures inside the virtual machine of the cloud. The storage facility that characterizes this simulation framework is a SAN of 500 TB. On top of the system, the OpenNebula 2.6 framework (http://www.opennebula.org/) is installed. This means that, at a time, the cluster is able to alternatively host 10 instances of type E, or 20 of D, or 40 of C or any feasible combination of them.

## 6. Simulation Results and Discussion

In the following, the three so-called zone allocation strategies, FE, SR, and MO, are compared in relation to the utilization, revenue, and user satisfaction metrics. To provide a more exhaustive picture about the ability of our algorithm to respond to different operating conditions, we first analyzed its behavior with respect to a basic scenario in which a uniform distribution between PZ and AZ requests has been simulated. We then varied the fifty-fifty composition of the arriving requests by alternatively considering a 75% versus 25% distribution of PZ (i.e., frequent users) and AZ (i.e., sporadic users). From this analysis, we then considered the impact on expected results caused by variations of the system configuration by upgrading the number of private nodes of 50% with respect to the basic scenario. In all cases, a large number of iterations have been carried out in order to ensure meaningfulness of simulated measurements. In particular, for more than 100 iterations, we have not gotten appreciable differences in the obtained results. The value of parameter *Q* for the SR strategy has been set to 50% (e.g. half the private resources) for every scenario considered. All simulations take place in a timeframe of one year, and a deadline of 48 hours was set only for AZ requests.

### 6.1. Basic Scenario

**Figure 4:**
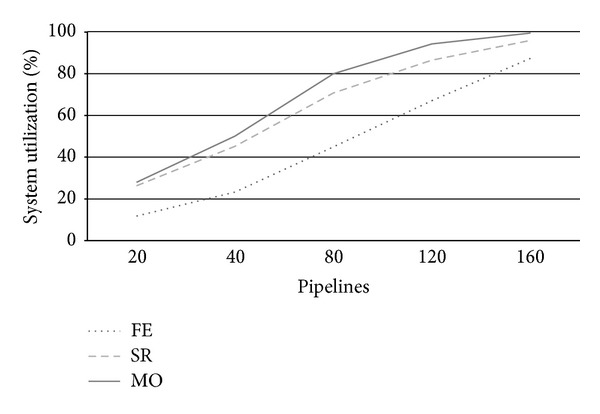
Private zone utilization *U* at varying arrival rates.

Figure 4 reports system utilization at varying number of arriving pipeline requests (*λ*). Utilization is a useful system measure that allows clarifying the behavior of each strategy at increasing system workloads according to the other metrics under analysis. From Figure 4, we see that for a number of pipelines around 80, FE strategy uses less than 50% of system resources, while SR and particularly MO take busy up to 80% system resources. This fact is clearly due to the conservative nature of FE, which uses private servers only to execute PZ requests, thus leading several resources idle. By contrast, SR and especially MO use up to all private cloud nodes to execute as more AZ requests as possible. We see that SR's utilization is always a 10% under MO one's due to its less aggressive use of system resources. Utilization further increases for higher load rates reaching almost saturation for *λ* = 120. When a system approximates saturation, its performance drastically worsens and it is no more able to adequately respond to user expectations. This situation impacts both on CB revenues and customers satisfaction. 

**Figure 5:**
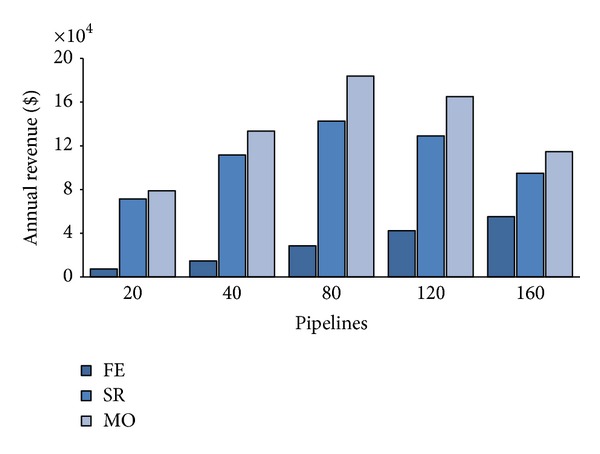
Annual CB's revenue.

Let us examine now the CB annual revenue.  Figure 5 shows the expected annual average revenue according to different values of the portion *Q* of the private cloud servers reserved for PZ requests at varying *λ*. For a number of submitted pipelines less than 80, we can notice linear revenue rising proportionally to *λ* increases for all values of the three policies. While for FE, this trend is maintained also for *λ* > 80 and both SR and MO revenues flex as the system gets closer to saturation. Notwithstanding this discontinuous behavior, MO policy allows higher revenue than the other two for all load rates. This fact is due to MO ability of accepting a greater number of AZ requests respect than SR and FE. The latter, in particular, neglects any AZ request, thus, renouncing to cash their 6-times greater price per VM (see Table 4), and the gap amongst FE and the other two strategies, shown by Figure 5, accounts for the revenue achieved by accepting sporadic customers pipelines (i.e., AZ ones).

The reason of not choosing always MO as unique allocation policy is due to its progressive inability of satisfactorily responding to customers' expectations. 

**Figure 6:**
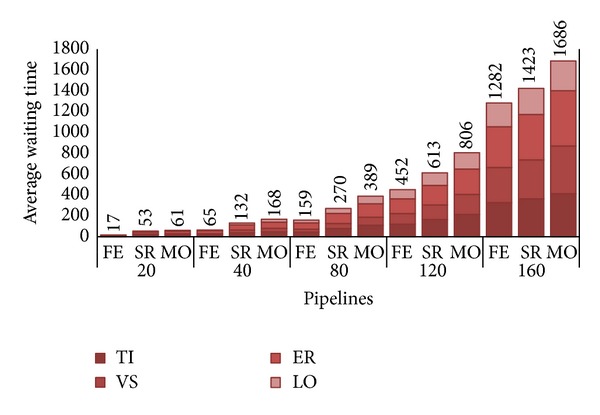
Average waiting times, in hours, per pipeline.

Let us look at the other metric, the user satisfaction. As explained in Section 5, to appreciate the variation of that measure, each histogram in Figure 6 depicts, for each *λ*/strategy pair, the average waiting time *W* before a PZ pipeline (i.e., composed of distinct PZ requests TI, VS, ER, and LO) is completely assigned to the private cluster. We are not interested here in examining waiting time of AZ requests, as they are always executed by the deadline agreed with the customers; that is, as said, if no space is found on private zone AZ are allocated to the public one as soon as deadline expires. 

The waiting time does not include execution time for each request but just reports the “wasted” time before a PZ request is taken in charge by the private cloud. It is important to note that, in the given example of a DD pipeline, research groups do not submit pipelines at once, that is, as a unitary workflow of (upmost) four requests, but the result of one operation determines if the following one will be executed or not. For this reason the reported value has to be intended as an estimate of the possible delay that has to be added both to the effective execution time as well as to the offline time required to examine the results of each single submission and, in case of success, the possible submission of the successive operation of the pipeline.

While in our simulations we got an average pipeline execution of about 310 hours (e.g. 88 for TI, 12 for VS, 206 for ER, and 4 for LO), the response time (i.e., sum of execution time and waiting time) increases as the number of pipelines, with 80 pipelines we have about half the waiting time with respect to execution time for FE (i.e., 159 hours), up to a waiting time exceeding the execution time for MO. With system approaching saturation, it is easy to observe how the waiting time becomes the major delaying factor of the chain, greatly penalizing every submission request. An obvious solution to bypass this issue is to upgrade the private zone capacity by augmenting the number of nodes. 

### 6.2. Alternative Scenarios

Before examining possible advantages of a (certainly) costly upgrade of the CB asset, it is important to analyze the impact of different distribution of users (i.e., sporadic and frequent) on the system behavior.

**Figure 7:**
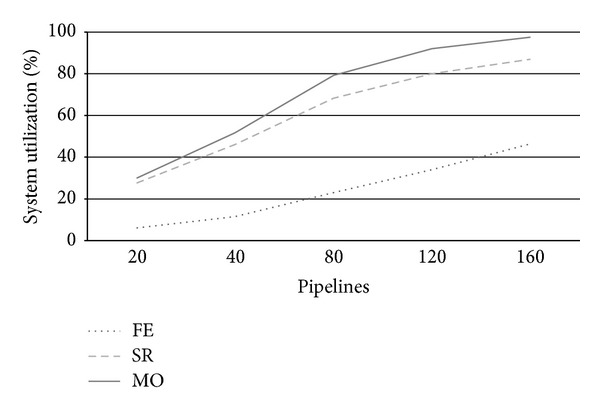
Private zone utilization *U* with 75% sporadic users.

**Figure 8:**
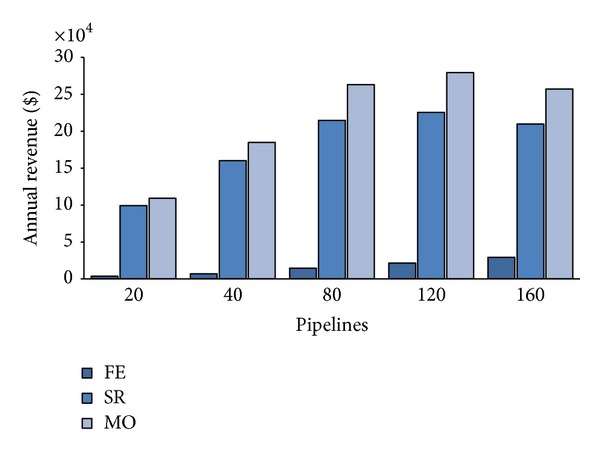
Annual CB's revenue with 75% sporadic users.

**Figure 9:**
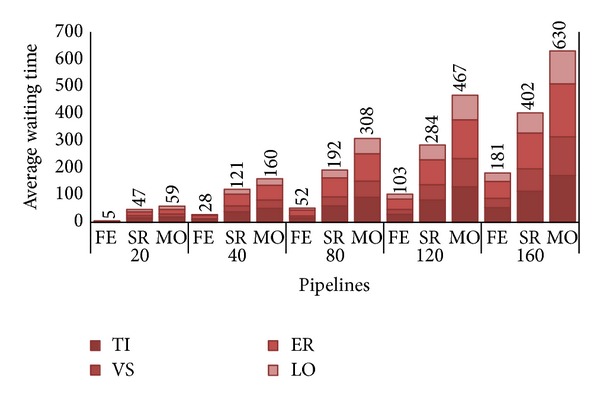
Average waiting times per pipeline with 75% sporadic users.

Figures 7, 8, and 9, respectively, depict the results of utilization *U*, annual revenue and average waiting time *W* of the three allocation strategies when the number of sporadic users is 75% of the total workload.

Having lowered the number of PZ requests, we see from Figure 7 that utilization greatly decreases in particular for FE that, at *λ* = 80, uses just 23% of private server against a 45% of the basic scenario. This fact directly impacts on the average waiting time that decreases to 52 hours from the 159 hours required in the fifty-fifty case. From Figure 9, we also see that, with just 25% of frequent users, FE allows more than acceptable waiting times also at higher workload (i.e., *W* = 181 at *λ* = 160). Quite sadly, Figure 8 shows that having reduced the number of PZ requests of a 50% also halved revenues of FE. Notwithstanding utilization for SR and MO is not dramatically reduced with respect to the basic scenario, we can appreciate considerable revenue increases both for SR than MO. This fact is reasonably explained by the fact that, in this scenario, the higher number of AZ requests, which pay more with respect to the PZ ones, allows greater revenues than before (e.g. MO achieved about $80,000 more).

Furthermore, the reduced number of PZ, that have to be mandatorily executed on the private cloud, allows to lower the average waiting times both for SR and MO. By comparing Figures 9 and 6, we notice that at *λ* = 80, SR required 192 hours in average to execute a PZ pipeline against the 270 of the basic scenario, while MO takes around 308 hours, respectively, to the previous 389. 

**Figure 10:**
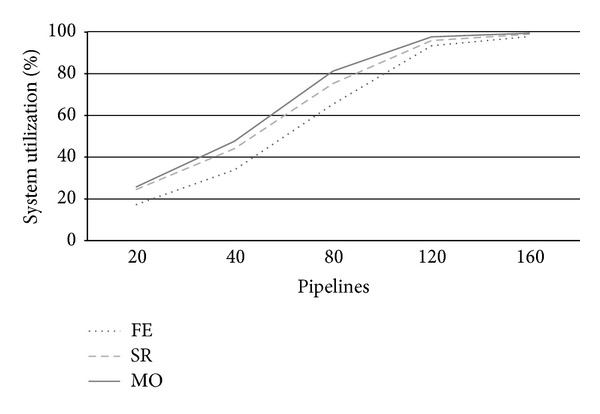
Private zone utilization *U* with 75% frequent users.

**Figure 11:**
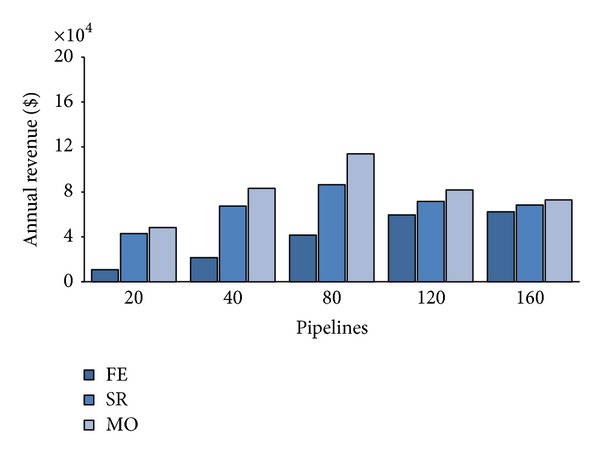
Annual CB's revenue with 75% frequent users.

Let us consider the opposite scenario, 75% of frequent users against 25% of sporadic ones. Not surprisingly, all the metrics considered show the worst performance also with respect the basic scenario. The only exception is the revenue achieved by FE, which is now 50% higher than the basic scenario consistently with the 50% more of PZ requests. Looking at utilization, we see from Figure 10 that the higher number of PZ requests quickly pushes the system near saturation; FE is about 65% at *λ* = 80, and both SR and MO approximate 100% at 120 workloads. Moreover, this kind of requests pay poorly to the CB (see Table 4) as shown in Figure 11; thus, we have a dramatic revenue decrease both for SR and MO which have to renounce to the higher revenue of an AZ pipeline with respect to a PZ one (e.g. MO's revenue is about $70,000 less than before).

**Figure 12:**
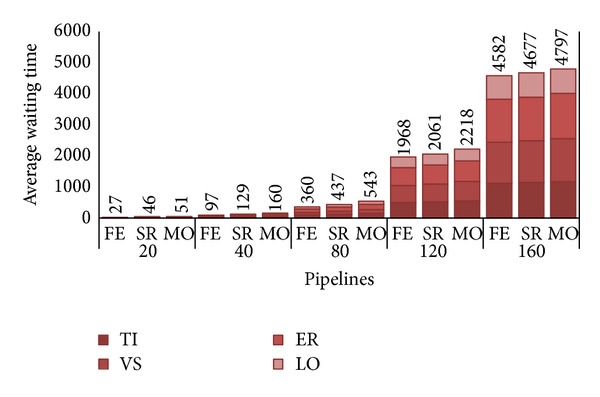
Average waiting times per pipeline with 75% frequent users.

Figure 12 depicts the extreme degradation of waiting times. The increase of *W* is essentially due the majority of requests that have to be strictly executed in-house. Just 25% of the overall arriving workload can now take place on the public cloud, while all the others (i.e., PZ ones) have to wait for some private resource being free. The great difference with respect to the other two scenarios, particularly the previous one, is the overwhelming abundance of PZ requests that tend to block the system, more than the AZ do. We remind that AZ requests leave the private zone to be allocated on the public one as soon as the deadline is reached. It seems reasonable to affirm that with the configuration of 10 nodes, this kind of distribution of requests strongly penalizes both the CB and its customers.

Once the attended composition of CB's customers is ascertained (or somehow estimated), the analysis of the upgraded configuration scenario makes more sense. In the following, we come back to consider the fifty-fifty customers' scenario and examine variations of the three metrics by a 50% system upgrading amounting for 15 private zone nodes.

**Figure 13:**
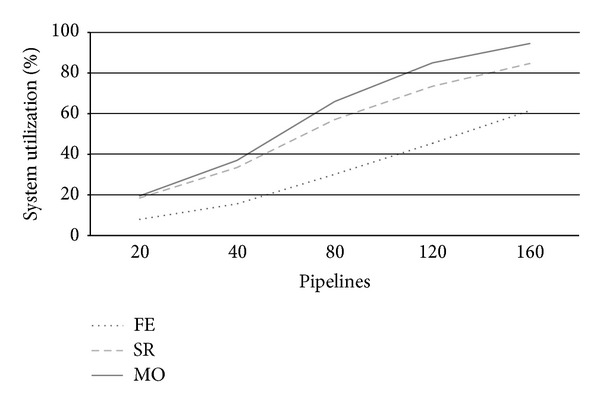
Private zone utilization *U*, with a 15-node system.

Figure 13 shows the reduction of resource utilization obtained by upgrading the system of 50%. This reduction is linear for FE: we have *U* = 30% instead of the previous 45%, and anyway appreciable for the other two strategies. Indeed, SR and MO are able to allocate internally a greater number of AZ requests than before; thus, system utilization improvement is less dramatic than FE but anyway significant: at *λ* = 80, SR accounts for *U* = 57%, and MO shows a *U* = 66% compared to previous *U* = 70% and *U* = 80% of the basic scenario.

**Figure 14:**
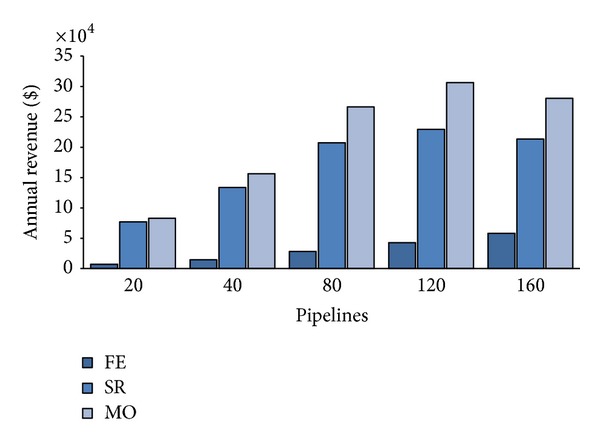
Annual CB's revenue with a 15-node system.

**Figure 15:**
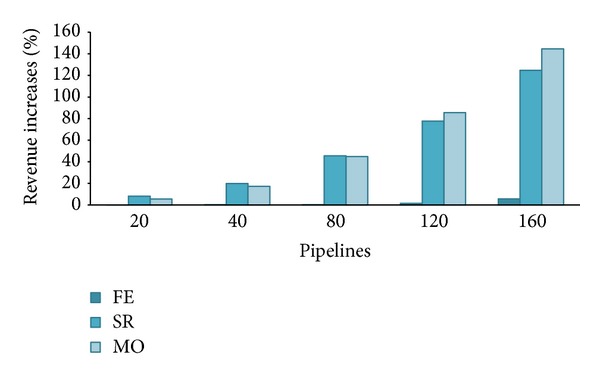
Percentage revenue increases with respect to the basic scenario (15 versus 10 nodes).

From Figure 14, we can appreciate the revenue increases that are achieved by SR and MO. To better highlight the magnitude of these increases, we reported in Figure 15 the percentage gain achieved with respect to the basic scenario. We see that nothing or less changed for FE; it begins to slightly improve just for *λ* > 80 (but for no more than 6% at *λ* = 160) when we have observed from Figures 4 and 6 that there was degradation in utilization that impacted on waiting time and hence in the number of PZ executed. With 50% more private nodes, system is now ready to satisfy a greater number of incoming requests. This major system capacity is better shown by the results achieved by SR and MO at *λ* = 80; they both measured a 45% revenue increase with respect to the basic scenario that further augments at higher loads. No surprisingly, the more computation availability, and the more AZ requests performed in-house instead turned to the public zone, the higher the revenues. Compared to Figure 5, we notice that now the flection of revenue for SR and MO begins at *λ* = 160 where system utilization approaches saturation (*U* = 84% and 94%); in the basic scenario, we had *U* = 86% and *U* = 95% before when *λ* = 120, which is exactly the 50% more of arriving pipelines.

**Figure 16:**
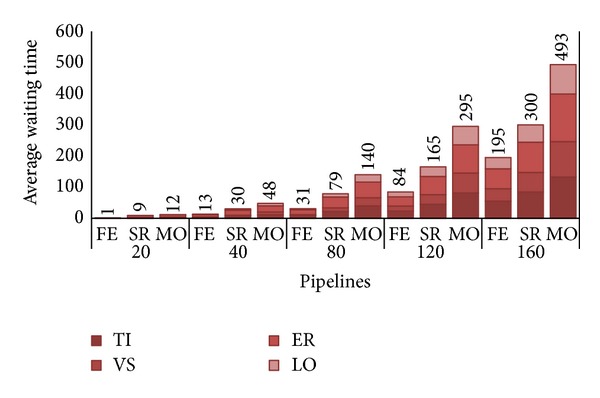
Average waiting times per pipeline with a 15-node system.

Having shifted the saturation to the higher loads, we see from Figure 16 that, at *λ* = 80, customers are awaiting for considerable less time than before. FE, SR, and MO have a reduction of 80%, 71%, and 64%, respectively, with FE and MO that show average waiting times around the 10% and 50% of the average execution time of a PZ pipeline. Until *λ* = 120, we notice acceptable waiting times for all three strategies with MO that approaches the execution time (i.e., 304 hours). As expected, *W* degrades nearby system saturation even if at least SR shows a waiting time quite similar to the pipeline execution time (i.e., 300 hours). This last observation leads us to conclude that a HC with a 15-node private zone, configured as specified in Section 5.2, seems able to yield considerable revenues for the CB without penalizing customers even in the case of great number of submitted pipelines. 

## 7. Conclusions and Future Works

Cloud computing is an emerging paradigm for supplying ICT infrastructures as a service also for e-Science applications, being able to provide high-level solutions in terms of compute capabilities and QoS. Cloud computing seems promising for a number of factors: it supports the easy deployment of services with respect to confirmed standard; the size of the available hardware allows to effectively deal with workload variations; preconfigured virtual machines can assist users in quickly performing their analyses. Cloud computing is able to provide clear advantages for small-medium laboratories working in the field of biotechnology, which typically do not have the possibility to invest a sufficient amount of time and money in creating and maintaining an in-house ICT infrastructure that suits the processing of the large amount of bioinformatics data that are nowadays produced. On the other hand, besides costs and QoS, security and customizability are critical points to consider when choosing ICT providers. This situation makes the cloud computing market an appealing opportunity for cloud brokers, a specialization of broker entities present also in SOA, despite the presence of leading companies as competitors. 

In this paper, we presented a brokering system for hybrid clouds and we analyzed some economic and practical aspects of exploiting cloud computing for the *in silico* drug discovery by studying the performance of three job-scheduling strategies (i.e., FE, SR, and MO). Based on the reservation of a quota of private cloud resources, the brokering tool manages the allocation of users' requests towards the public or the private cloud zone depending on the QoS expectations and the workload of in-house resources. Simulation results examined the response of the various policies in terms of utilization and user satisfaction as well as provided an analysis of economics advantages for the two stakeholders of the scenario: the CB and the small-medium biotechnological laboratories.

From the results analysis follows that, although MO is certainly the best policy as regards CB revenue, it strongly penalizes customers, especially at medium-high load rates, due to its longer waiting times. For these reasons, SR, which follow a less greedy approach, may represent a good compromise between CB economic goals and users' satisfaction. Moreover, we observed in Section 6.2 the importance of the composition of the arriving customers. Sporadic customers are preferable from the CB point of view as they allow greater revenues (for SR and MO) meanwhile significantly reducing waiting times. Obviously from the sporadic customer point of view, the choice to become a frequent one, thus obtaining considerable better prices for an entire pipeline execution, has accurately been evaluated based on the number of pipelines the group foresees to yearly submit to the cloud. We also noticed that to better respond to an increasing number of pipelines, a system upgrade could certainly be beneficial for all the metrics, and the strategies considering this choice have to be carefully evaluated by the CB in the light of the effective number and composition of the expected pipelines.

Further directions of this study will consider energy-savings methods which integrate VM migration mechanisms and take into account the energy-performance trade-off.

## Figures and Tables

**Table 1:** Characteristics of the operations of the drug discovery pipeline.

	TI	VS	ER	LO
*Functional *				
Number ofcores	≥16	≥8	≥32	≥4
Network	—	—	Infiniband	—
RAM (GB)	≥32	≥16	≥64	≥16
HDD (GB)	—	≥500	≥1000	—
Reference Amazon EC2^1^ virtual cluster	20 × D	10 × 2 × D	1–10 × 2 × D	1 × C
*Non functional *				
Security	Low	Medium	Medium	High
Availability/Resiliency	Low	Medium	Medium	Low
*Operations parameters *				
Input data and size	10 K–100 K Parameter simulations	5 K–10 K Compounds	1–10 Simulations 50 K–250 K Atoms 5–300 Nanoseconds	5–10 Compounds
Service demand time (*μ*_*k*_) with respect to the reference Amazon EC2 virtual cluster	16–160 h	8–16 h	2–1200 h	1–6 h
Arrival rate (*λ*_*k*_) per year per research group	4	3/4	2/3	1/2
*Packages *				
Commercial	MATLAB	FlexX, Glide, ICM	AMBER, CHARMM	MacroModel, Medstere
Free/open source	R	Dock, Autodock	GROMACS, NAMD	Omega

^1^See Table 2.

**Table 2:** Characteristics of the considered Amazon EC2 instance types.

Amazon EC2 instance types	RAM	EC2 compute unit	Storage	On Demand instances price/h	Heavy utilization reserved instances price/h
A: High-Memory Double eXtra Large	34.2 GB	13	850 GB	$0.9	$0.176
B: High-Memory Quadruple XL	68.4 GB	26	1690 GB	$1.8	$0.352
C: Cluster Compute Quadruple XL	23 GB	33.5	1690 GB	$1.3	$0.297
D: Cluster Compute Eight XL	60.5 GB	88	3370 GB	$2.4	$0.361

**Table 3:** Commercial and open source cloud broker Platforms.

	Cloud service	Users^1^	CI^2^	Type of X as a Service	QoS/SLA	Billing/ Bench.	SW^3^
Zimory^4^	Management system	CB, SP, CC	All	All	Security, resiliency/Y		APIs
ComputeNext^5^	Service brokerage	SP, CC	—	All (I)	—/Y	Y/Y	APIs
CompatibleOne^6^	Cloud Broker	All	All	All	Security/Y		OS APIs
Gravitant cloudMatrix^7^	Services brokerage	CB, SP, CC	All	All	Security/Y	Y/Y	APIs
enStratius^8^	Management system	CB, SP, CC	All	S, I	Security	Y	APIs
RightScale myCloud^9^	Management system	SP, CC	All (H)	S, P	Security	Y	APIs
Scalr^10^	Management system	SP, CC	All	All			[OS] APIs
Standing Cloud^11^	Marketplace	All	PB	All			
Artisan Infrastructure^12^	IaaS provider	SP		All	Security, resiliency/Y		
StratusLab^13^	IaaS distribution	CB, SP	—	I	—	—	OS APIs
Contrail^14^	Components	CP, CB, SP	All	P, I	Security, reliability/Y		OS APIs
RESERVOIR^15^	Federated clouds management	CB, SP	H	I	—/Y		OS APIs
MOSAIC^16^	Component framework	SP, CB	H, P	All	Security/Y	—/Y	OS APIs

^1^Cloud Provider, Cloud Broker, Service Provider, Cloud Consumer.

^2^Cloud Infrastructure: PuBlic, Private, Hybrid.

^3^License: Open Source.

^4^ http://www.zimory.com.

^5^ https://www.computenext.com.

^6^ http://www.compatibleone.org.

^7^ http://www.gravitant.com.

^8^ http://www.enstratius.com.

^9^ http://www.rightscale.com.

^10^ http://www.scalr.com.

^11^ http://www.standingcloud.com.

^12^ http://www.artisaninfrastructure.com.

^13^ http://stratuslab.eu.

^14^ http://contrail-project.eu.

^15^ http://www.reservoir-fp7.eu.

^16^ http://www.mosaic-cloud.eu.

**Table 4:** VM configurations and hourly provisioning price per type of requests.

Instance type	RAM	Cores	Storage	Operation	AZ price/h	PZ price/h
C	32 GB	8	1690 GB	LO	$1.3	$0.297
D	64 GB	16	3370 GB	TI	$2.4	$0.361
E	128 GB	32	1-2 TB	VS, ER	$4.8	$0.722

## References

[1] Davie P (2010). Cloud computing: a drug discovery game changer?. *Innovations in Pharmaceutical Technology*.

[2] Krampis K, Booth T, Chapman B (2012). Cloud BioLinux: pre-configured and on-demand bioinformatics computing for the genomics community. *BMC Bioinformatics*.

[3] Angiuoli SV, Matalka M, Gussman A (2011). CloVR: a virtual machine for automated and portable sequence analysis from the desktop using cloud computing. *BMC Bioinformatics*.

[4] Ferretti S, Ghini V, Panzieri F, Pellegrini M, Turrini E QoS-aware clouds.

[5] Ahuja SP, Mani S (2012). The state of high performance computing in the cloud. *Journal of Emerging Trends in Computing and Information Sciences*.

[6] Salomoni D, Italiano A, Ronchieri E (2011). WNoDeS, a tool for integrated Grid and Cloud access and computing farm virtualization. *Journal of Physics*.

[7] Three Types of Cloud Brokerages Will Enhance Cloud Services. http://www.gartner.com/DisplayDocument?ref=g_search&id=973412&subref=simplesearch.

[8] Buyya Q, Broberg J, Goscinski AM (2011). *Cloud Computing: Principles and Paradigms*.

[9] Grover S, Apushkin MA, Fishman GA (2006). Topical dorzolamide for the treatment of cystoid macular edema in patients with retinitis pigmentosa. *The American Journal of Ophthalmology*.

[10] Von Itzstein M, Wu W-Y, Kok GB (1993). Rational design of potent sialidase-based inhibitors of influenza virus replication. *Nature*.

[11] Terrett NK, Bell AS, Brown D, Ellis P (1996). Sildenafil (Viagra(TM)), a potent and selective inhibitor of type 5 CGMP phosphodiesterase with utility for the treatment of male erectile dysfunction. *Bioorganic and Medicinal Chemistry Letters*.

[12] Goodgame JC, Pottage JC, Jablonowski H (2000). Amprenavir in combination with lamivudine and zidovudine versus lamivudine and zidovudine alone in HIV-1-infected antiretroviral-naive adults. *Antiviral Therapy*.

[13] Song CM, Lim SJ, Tong JC (2009). Recent advances in computer-aided drug design. *Briefings in Bioinformatics*.

[14] Butcher EC, Berg EL, Kunkel EJ (2004). Systems biology in drug discovery. *Nature Biotechnology*.

[15] McInnes C (2007). Virtual screening strategies in drug discovery. *Current Opinion in Chemical Biology*.

[16] Ghosh S, Nie A, An J, Huang Z (2006). Structure-based virtual screening of chemical libraries for drug discovery. *Current Opinion in Chemical Biology*.

[17] Durrant JD, McCammon JA (2011). Molecular dynamics simulations and drug discovery. *BMC Biology*.

[18] D’Ursi P, Chiappori F, Merelli I, Cozzi P, Rovida E, Milanesi L (2009). Virtual screening pipeline and ligand modelling for H5N1 neuraminidase. *Biochemical and Biophysical Research Communications*.

[19] Chiappori F, Merelli I, Milanesi L, Marabotti A (2013). Static and dynamic interactions between GALK enzyme and known inhibitors: guidelines to design new drugs for galactosemic patients. *European Journal of Medicinal Chemistry*.

[20] Bugatti A, Giagulli C, Urbinati C (2013). Molecular interaction studies of HIV-1 matrix protein p17 and heparin: identification of the heparin-binding motif of p17 as a target for the development of multitarget antagonists. *Journal of Biological Chemistry*.

[21] Cichero E, Ligresti A, Allar M (2011). Homology modeling in tandem with 3D-QSAR analyses: a computational approach to depict the agonist binding site of the human CB2 receptor. *European Journal of Medicinal Chemistry*.

[22] Merelli I, Cozzi P, D’Agostino D, Clematis A, Milanesi L (2011). Image-based surface matching algorithm oriented to structural biology. *IEEE/ACM Transactions on Computational Biology and Bioinformatics*.

[23] Mosca E, Barcella M, Alfieri R, Bevilacqua A, Canti G, Milanesi L (2012). Systems biology of the metabolic network regulated by the Akt pathway. *Biotechnology Advances*.

[24] Plewczynski D, Hoffmann M, Von Grotthuss M, Ginalski K, Rychewski L (2007). In silico prediction of SARS protease inhibitors by virtual high throughput screening. *Chemical Biology and Drug Design*.

[25] Lew W, Chen X, Kim CU (2000). Discovery and development of GS 4104 (oseltamivir): an orally active influenza neuraminidase inhibitor. *Current Medicinal Chemistry*.

[26] Wolf A, Shahid M, Kasam V, Ziegler W, Hofmann-Apitius M (2010). In silico drug discovery approaches on grid computing infrastructures. *Current Clinical Pharmacology*.

[27] Lang PT, Brozell SR, Mukherjee S (2009). DOCK 6: combining techniques to model RNA-small molecule complexes. *RNA*.

[28] Morris GM, Ruth H, Lindstrom W (2009). Software news and updates AutoDock4 and AutoDockTools4: automated docking with selective receptor flexibility. *Journal of Computational Chemistry*.

[29] Cross SSJ (2005). Improved FlexX docking using FlexS-determined base fragment placement. *Journal of Chemical Information and Modeling*.

[30] Friesner RA, Murphy RB, Repasky MP (2006). Extra precision glide: docking and scoring incorporating a model of hydrophobic enclosure for protein-ligand complexes. *Journal of Medicinal Chemistry*.

[31] An J, Totrov M, Abagyan R (2005). Pocketome via comprehensive identification and classification of ligand binding envelopes. *Molecular and Cellular Proteomics*.

[32] Irwin JJ, Shoichet BK (2005). ZINC: a free database of commercially available compounds for virtual screening. *Journal of Chemical Information and Modeling*.

[33] Lipinski CA, Lombardo F, Dominy BW, Feeney PJ (2001). Experimental and computational approaches to estimate solubility and permeability in drug discovery and development settings. *Advanced Drug Delivery Reviews*.

[34] Huey R, Morris GM, Olson AJ, Goodsell DS (2007). Software news and update a semiempirical free energy force field with charge-based desolvation. *Journal of Computational Chemistry*.

[35] Alonso H, Bliznyuk AA, Gready JE (2006). Combining docking and molecular dynamic simulations in drug design. *Medicinal Research Reviews*.

[36] Malmstrom RD, Watowich SJ (2011). Using free energy of binding calculations to improve the accuracy of virtual screening predictions. *Journal of Chemical Information and Modeling*.

[37] Ekins S, Mestres J, Testa B (2007). *In silico* pharmacology for drug discovery: applications to targets and beyond. *British Journal of Pharmacology*.

[38] Gedeck P, Lewis RA (2008). Exploiting QSAR models in lead optimization. *Current Opinion in Drug Discovery and Development*.

[39] Lee H-C, Salzemann J, Jacq N (2006). Grid-enabled high-throughput in silico screening against influenza a neuraminidase. *IEEE Transactions on Nanobioscience*.

[40] Case DA, Darden TA, Cheatham TE (2012). *AMBER 12*.

[41] Hess B, Kutzner C, van der Spoel D, Lindahl E (2008). GRGMACS 4: algorithms for highly efficient, load-balanced, and scalable molecular simulation. *Journal of Chemical Theory and Computation*.

[42] Phillips JC, Braun R, Wang W (2005). Scalable molecular dynamics with NAMD. *Journal of Computational Chemistry*.

[43] Brooks BR, Brooks CL, Mackerell AD (2009). CHARMM: the biomolecular simulation program. *Journal of Computational Chemistry*.

[44] Afgan E, Baker D, Coraor N, Chapman B, Nekrutenko A, Taylor J (2010). Galaxy CloudMan: delivering cloud compute clusters. *BMC Bioinformatics*.

[45] Chouvarine P, Cooksey AM, McCarthy FM (2012). Transcriptome-based differentiation of closely-related Miscanthus lines. *PLoS ONE*.

[46] Stein LD (2010). The case for cloud computing in genome informatics. *Genome Biology*.

[47] Schatz MC, Langmead B, Salzberg SL (2010). Cloud computing and the DNA data race. *Nature Biotechnology*.

[48] Kang L, Guo Q, Wang X (2012). A hierarchical method for molecular docking using cloud computing. *Bioorganic and Medicinal Chemistry Letters*.

[49] Garg V, Arora S, Gupta C (2011). Cloud computing approaches to accelerate drug discovery value chain. *Combinatorial Chemistry and High Throughput Screening*.

[50] Dai L, Gao X, Guo Y, Xiao J, Zhang Z (2012). Bioinformatics clouds for big data manipulation. *Biology Direct*.

[51] Alfieri, Arezzini R, Barone S The HPC Testbed of the Italian Grid Infrastructure.

[52] http://www.venus-c.eu/deliverables_year1/VENUS-C_D3.9.pdf.

[53] Kudtarkar P, DeLuca TF, Fusaro VA, Tonellato PJ, Wall DP (2010). Cost-effective cloud computing: a case study using the comparative genomics tool, roundup. *Evolutionary Bioinformatics*.

[54] Armbrust M, Fox A, Griffith R (2009). Above the clouds: a Berkeley view of cloud computing. *Technical Report*.

[55] Lee CA A perspective on scientific cloud computing.

[56] Prodan R, Wieczorek M, Fard HM (2011). Double auction-based scheduling of scientific applications in distributed grid and cloud environments. *Journal of Grid Computing*.

[57] Li J, Peng J, Zhang W (2011). A scheduling algorithm for private clouds. *Journal of Convergence Information Technology*.

[58] Garg SK, Yeo CS, Anandasivam A, Buyya R (2011). Environment-conscious scheduling of HPC applications on distributed Cloud-oriented data centers. *Journal of Parallel and Distributed Computing*.

[59] Ekanayake J, Gunarathne T, Qiu J (2011). Cloud technologies for bioinformatics applications. *IEEE Transactions on Parallel and Distributed Systems*.

[60] Roloff E, Birck F, Diener M, Carissimi A, Navaux POA Evaluating high performance computing on the windows azure platform.

[61] Quarati A, D'Agostino D, Galizia A, Mangini M, Clematis A (2012). Delivering cloud services with QoS requirements: an opportunity for ICT SMEs. *Economics of Grids, Clouds, Systems, and Services*.

[62] D'Agostino D, Galizia A, Clematis A, Mangini M, Porro I, Quarati A (2013). A QoS-aware broker for hybrid clouds. *Computing*.

[63] Law AM (2007). *Simulation Modeling and Analysis*.

[64] Menascé DA, Almeida VA, Dowdy LW (2004). *Performance By Design: Computer Capacity Planning By Example*.

[65] Nou R, Kounev S, Torres J (2007). Building online performance models of grid middleware with fine-grained load-balancing: a globus toolkit case study. *Formal Methods and Stochastic Models for Performance Evaluation*.

